# Supplementary material for: Genomic assessment of quarantine measures to prevent SARS-CoV-2 importation and transmission
Source: Nat Commun. 2022 Feb 23;13:1012. doi: 10.1038/s41467-022-28371-z (PMC8866425; doi:10.1038/s41467-022-28371-z)
Supplement: Supplementary file 1 — Supplementary Information [file 41467_2022_28371_MOESM1_ESM.pdf]

Genomic assessment of quarantine measures to prevent SARS-CoV-2 importation and transmission

Supplementary Figures

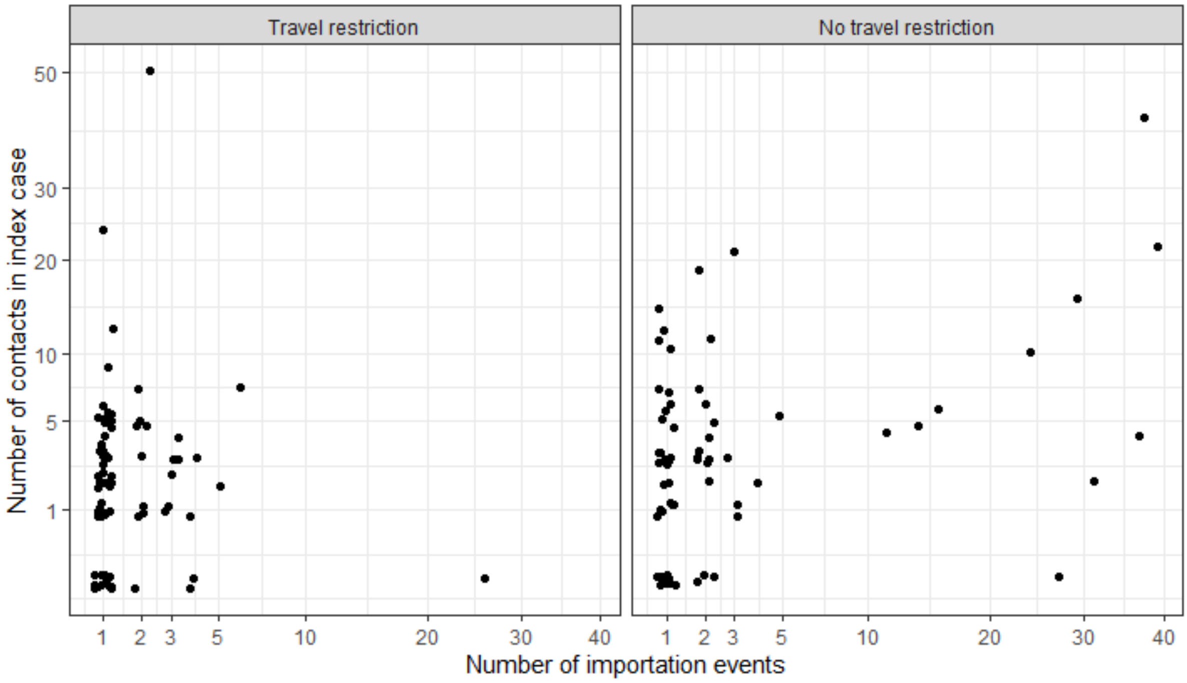

Supplementary Figure 1. The number of contacts of the index case in relation to the number of importation events. There was some evidence that imported cases with higher numbers of contacts for the index case gave rise to more cases in the subsequent month, which may be explained by the number of importations; there is a positive correlation (Spearmans rho=0.18, p=0.018) between the number of contacts reported by the index case and the number of independent importations of each genome

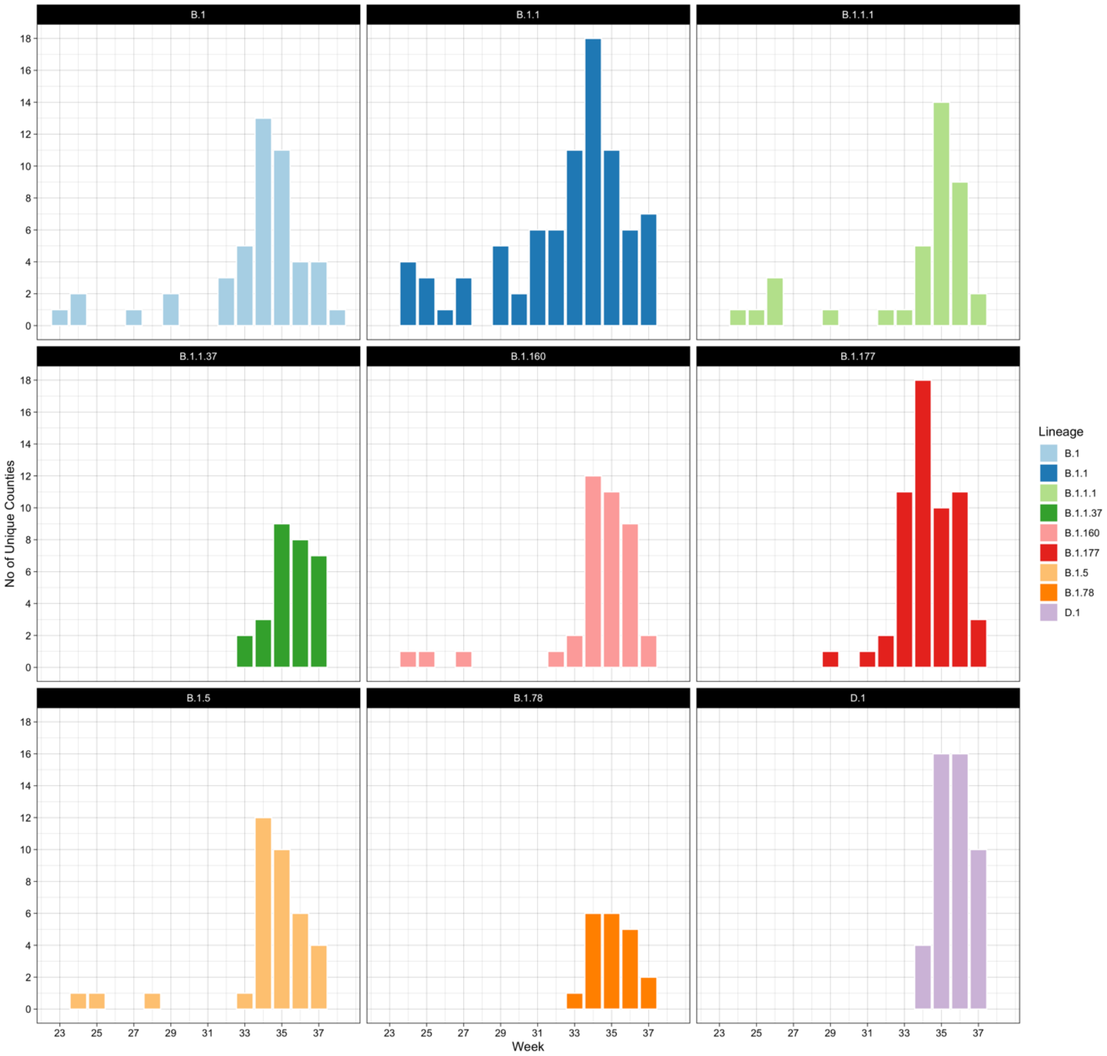

Supplementary Figure 2: The dispersion of importations of different lineages throughout England per week. This represents the top 9 global lineages versus the number of unique counties the lineage is found in, using the county provided by the case. The counties are the lieutenancies or ceremonial counties of which there are 48.

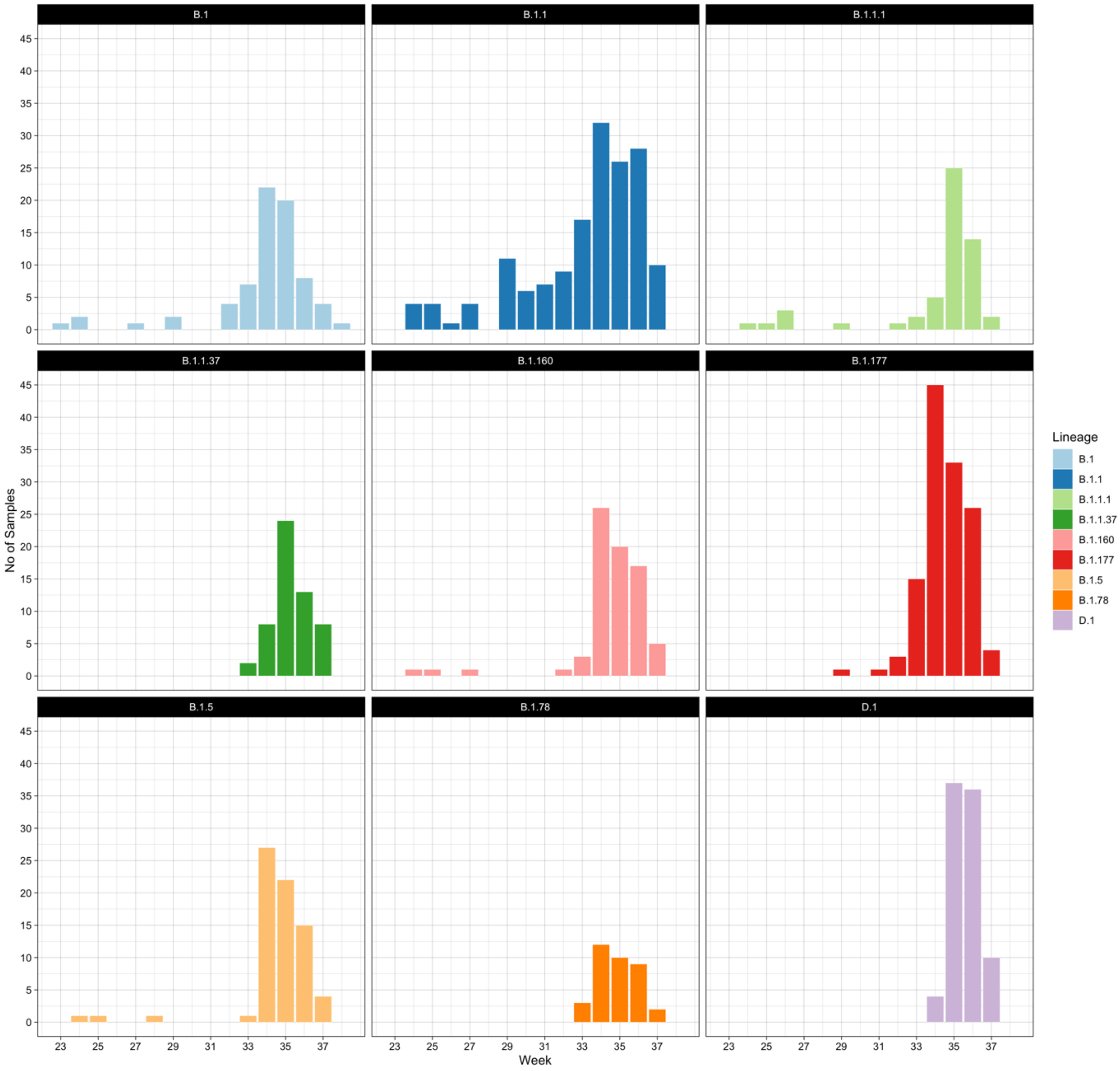

Supplementary Figure 3: The number of importations of each global lineage per week of 2020. This Figure represents the Top 9 global lineages.

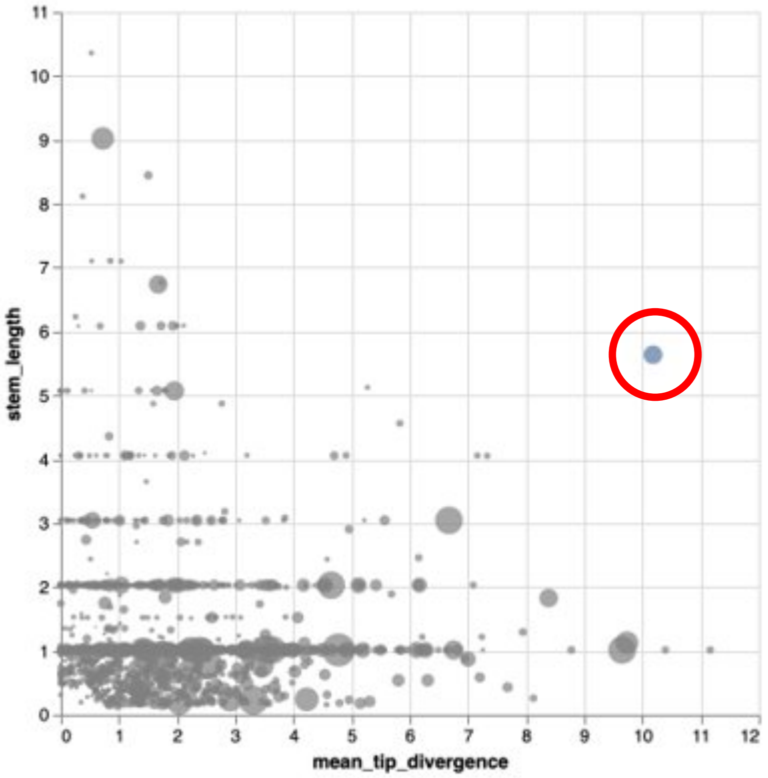

Supplementary Figure 4: Polecat cluster analysis with the likely travel-related cluster highlighted. The red circle highlights the divergent cluster which we subsequently demonstrated to be travel-related

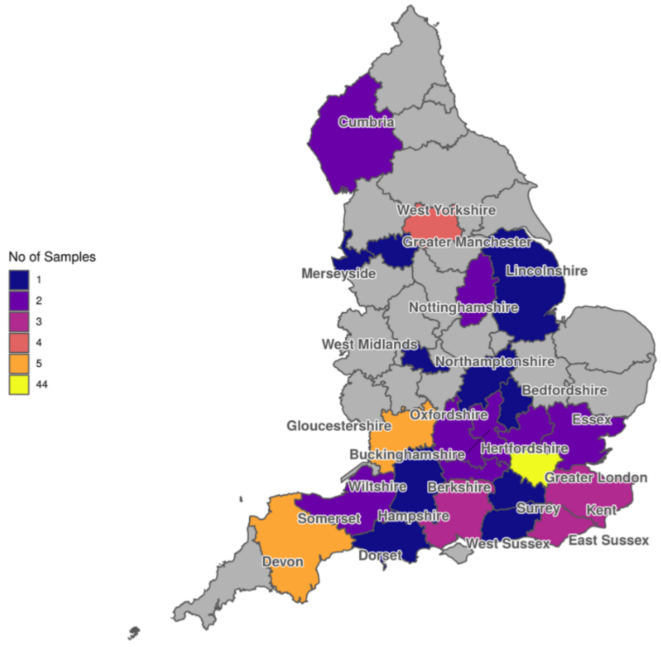

Supplementary Figure 5: No. of genomes of importations or their contacts of lineage UK1897 per county in England.

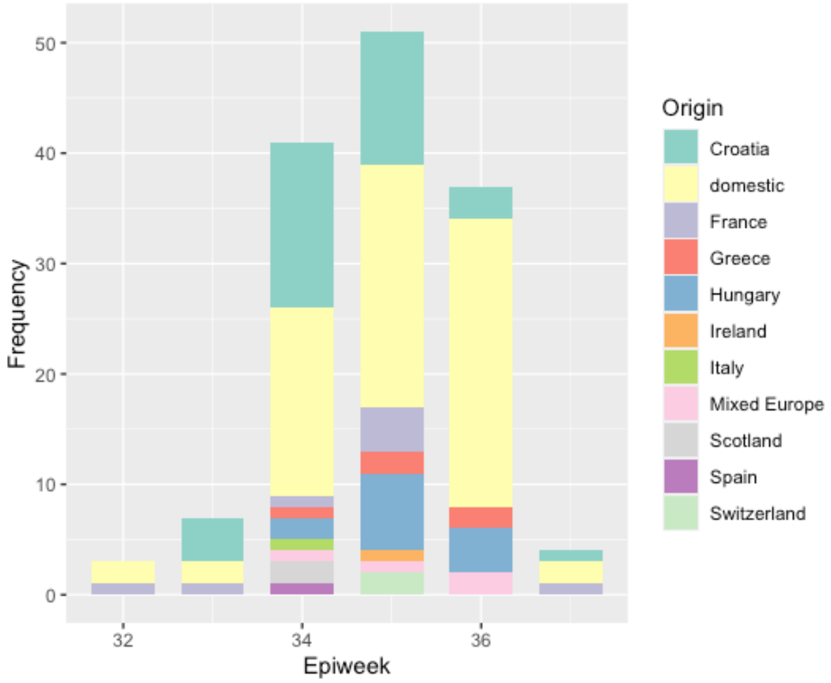

Supplementary Figure 6: Source country of SARS-CoV-2 acquisition for individuals identified within a suspected travel-related cluster highlighted by the Polecat tool, represented by epiweek

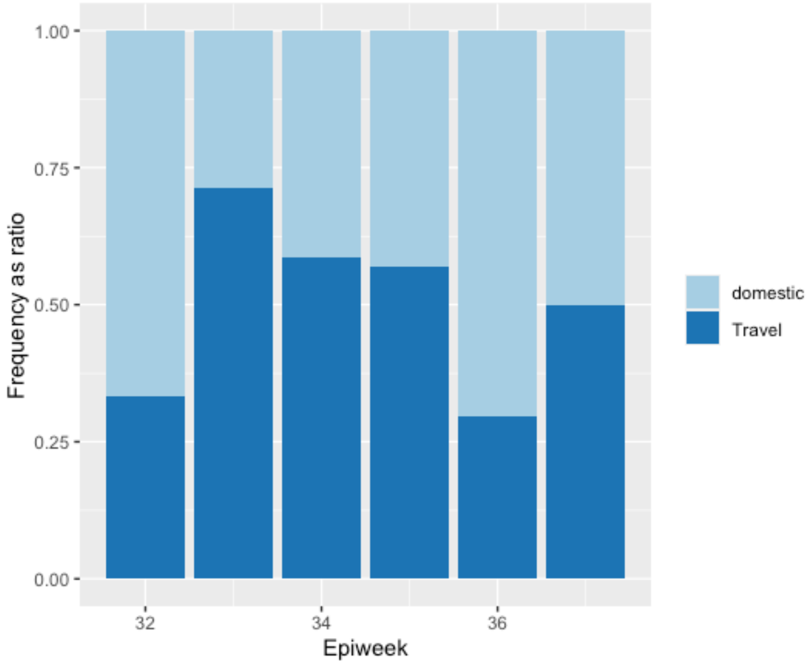

Supplementary Figure 7: Frequency of individuals identifying a travel or domestic source of SARS-CoV-2 acquisition within the suspected travel-related cluster of genomes highlighted by the Polecat tool, represented by epiweek

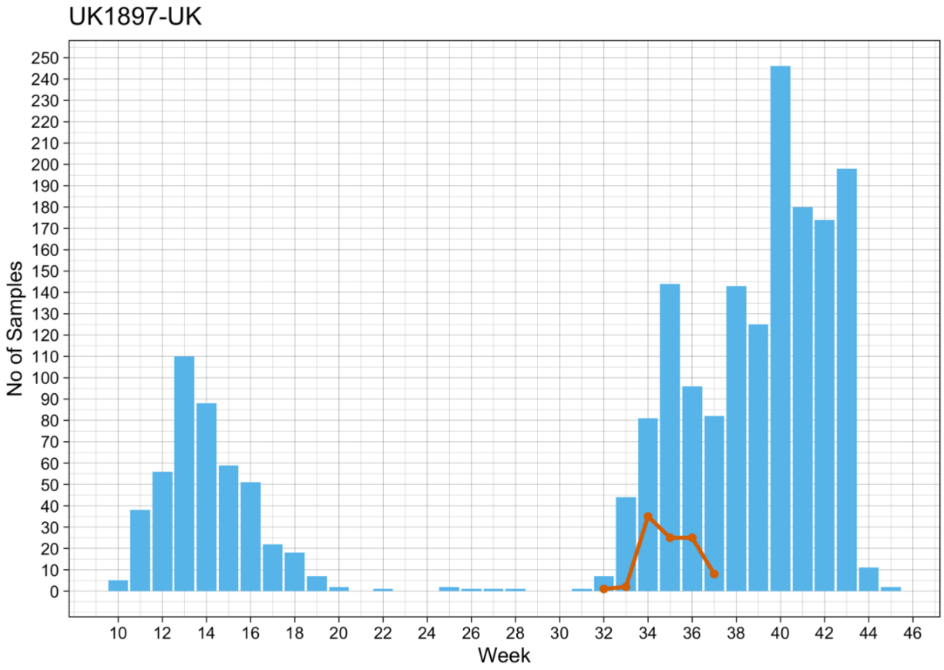

Supplementary Figure 8: UK1897 SARS-CoV-2 lineage in the United Kingdom by epiweek. The line (orange) is the number of genomes which are confirmed importations from the lineage UK1897 per week of 2020. The blue bars indicate the number of genomes of this lineage seen per week anywhere in the UK (including the importations).

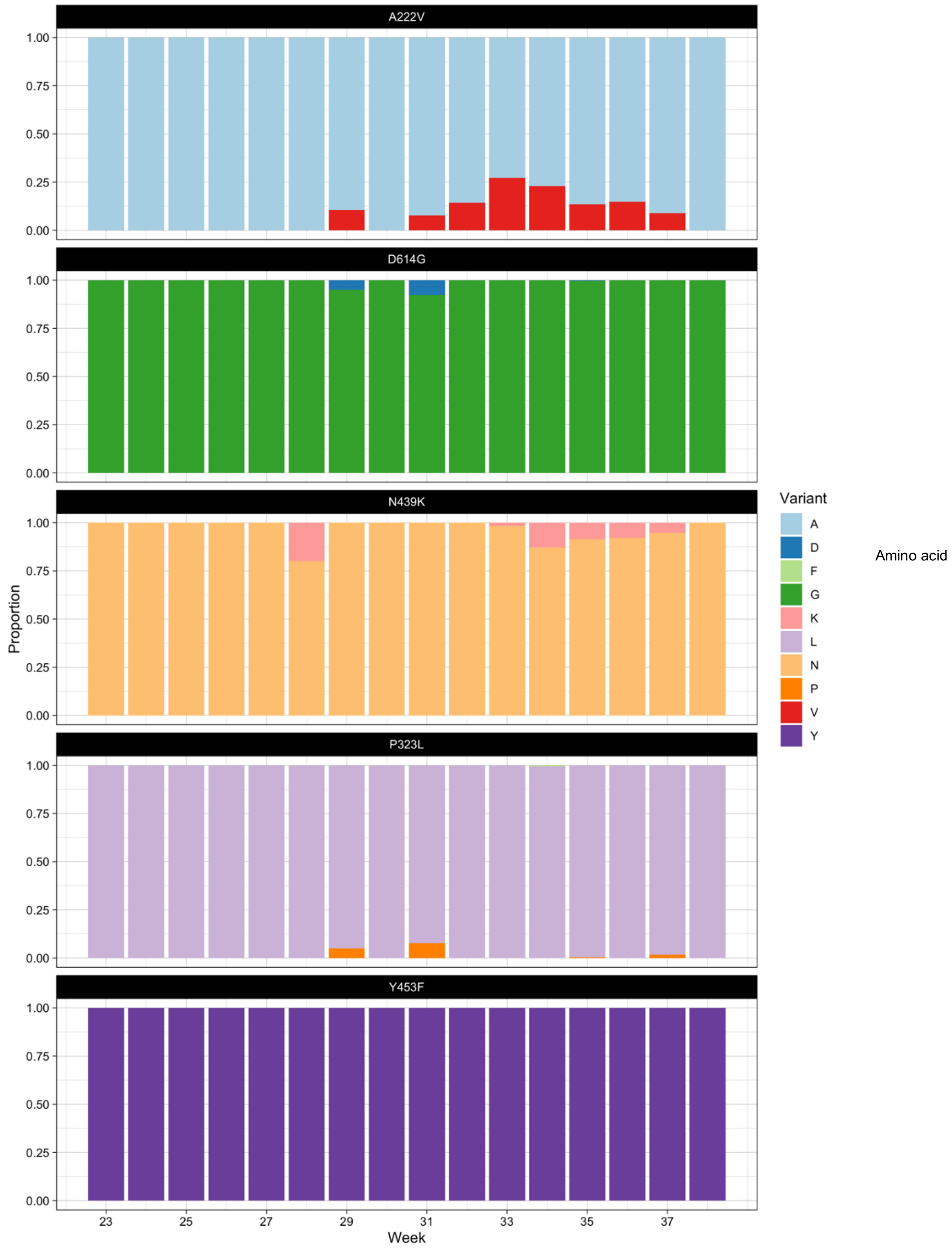

Supplementary Figure 9: The percentage of each major mutation observed per week in the imported genomes. Letters in the amino acid substitution nomenclature correspond to: A, alanine; D, aspartic acid; F, phenylalanine; G, glycine; K, lysine; L, leucine; N, asparagine; P, proline; V, valine; Y, tyrosine. The mutations are named as following: the letter preceding number (the amino acid site of substitution) represents the wild-type amino acid, the letter following the number is the observed amino acid in the sample ('a mutation', if different from the wild-type). The figure legend represents the observed amino acid at the site of interest, e.g. 'A' in the panel representing the A222V mutation shows cases observing alanine at site 222.

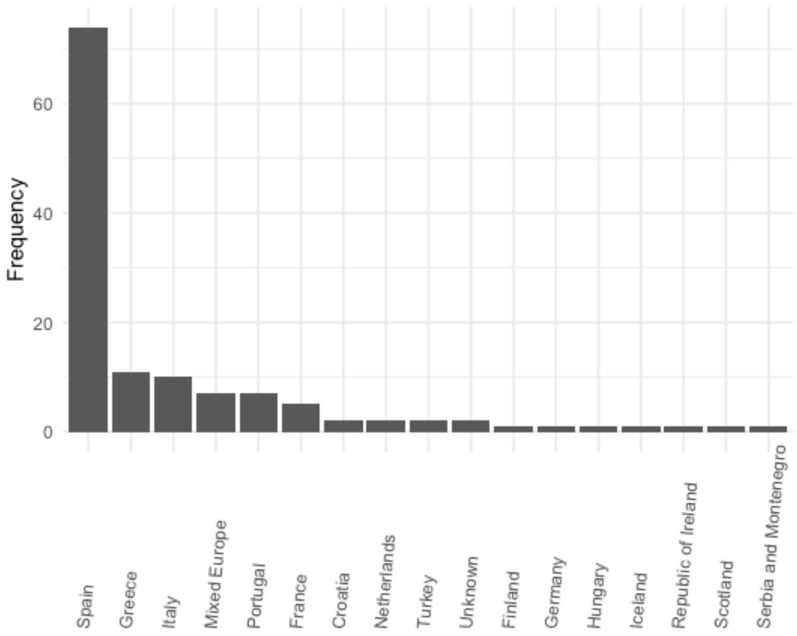

Supplementary Figure 10: Destination country of travel-related SARS-CoV-2 with the A222V variant identified, during the study period

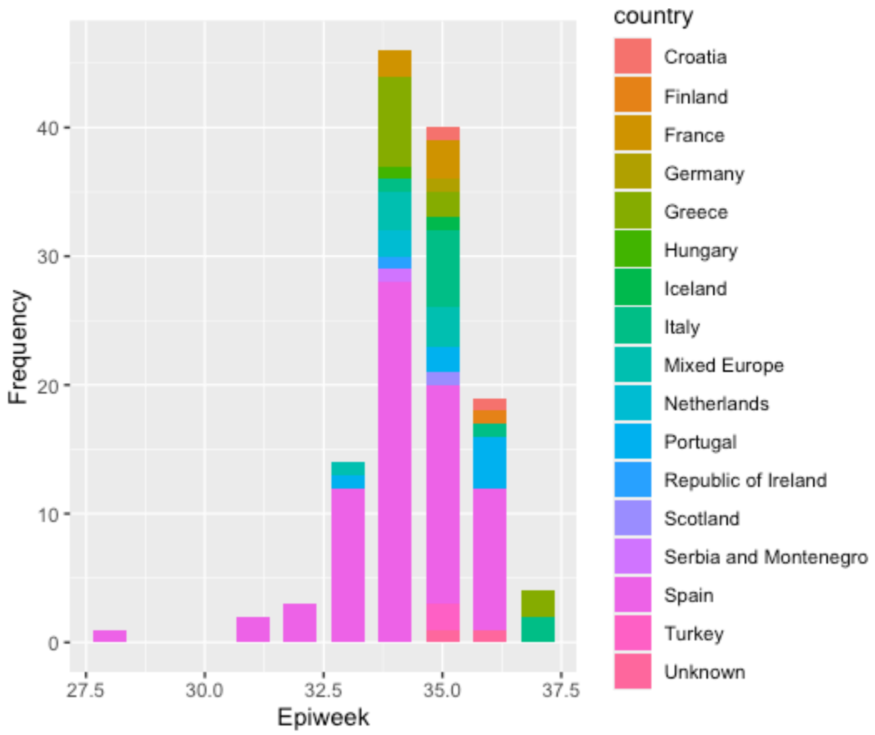

Supplementary Figure 11: Reported country of travel for cases with the A222V variant of SARS-CoV-2, imported over time

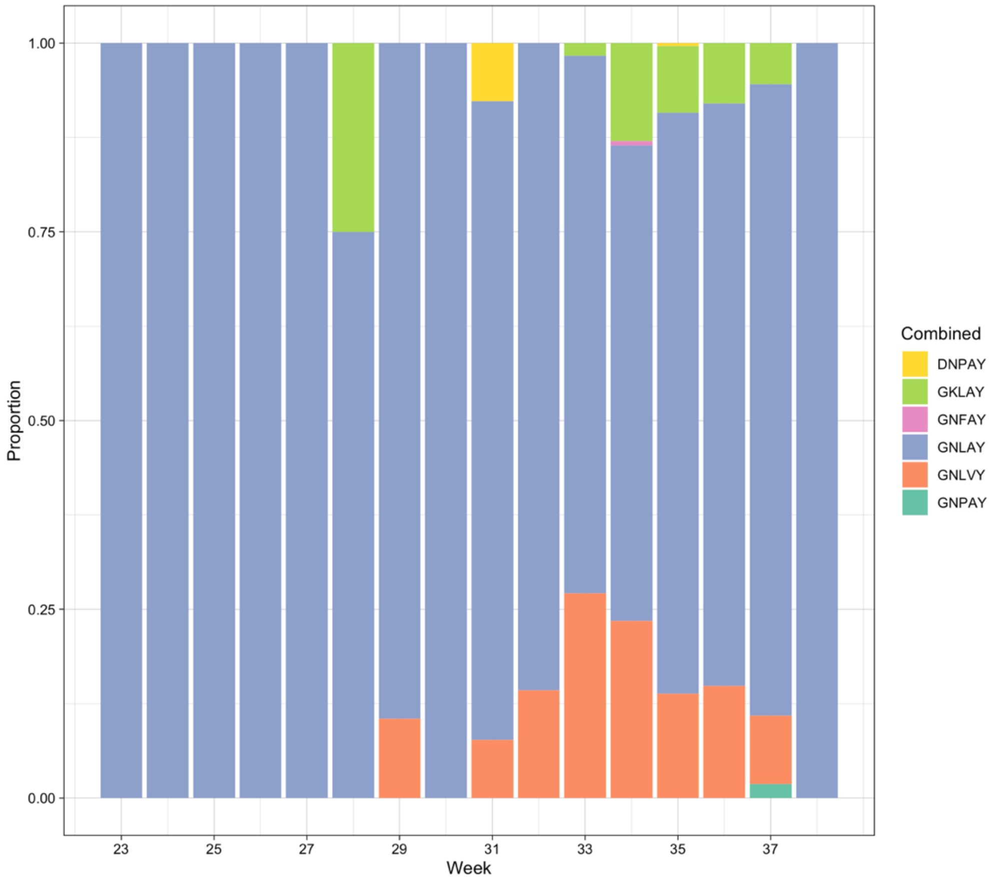

Supplementary Figure 12: Combination of SARS-CoV-2 mutations seen in imported SARS-CoV-2 genomes by epiweek. The combinations of co-occurring variants, where the variants are in the order: 1) D614G, 2) N439K,3) P323L, 4) A222V and 5) Y453F. Letters in the amino acid substitution nomenclature correspond to: A, alanine; D, aspartic acid; F, phenylalanine; G, glycine; K, lysine; L, leucine; N, asparagine; P, proline; V, valine; Y, tyrosine. The mutations are named as following: the letter preceding number (the amino acid site of substitution) represents the wild-type amino acid, the letter following the number is the observed amino acid in the sample ('a mutation', if different from the wild-type).

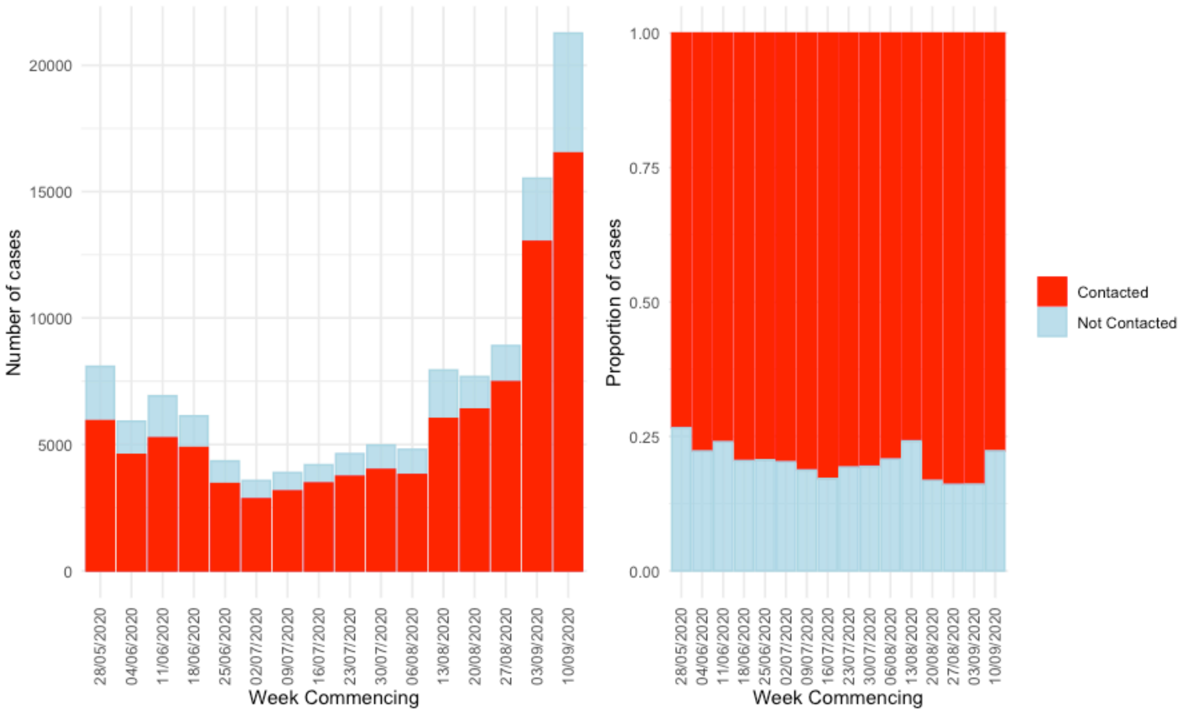

Supplementary Figure 13: SARS-CoV-2 cases successfully contacted by Test and Trace in England from 28/05/2020 to 14/09/2020. (a) Number of cases successfully contacted and the epidemic curve in England over the study period. **(b)** Proportion of cases successfully contacted over the study period. There is little observed change in the proportion of successful cases contacted over time. Note: Data is binned into weeks (7-day periods) with each week commencing on a Thursday[1]

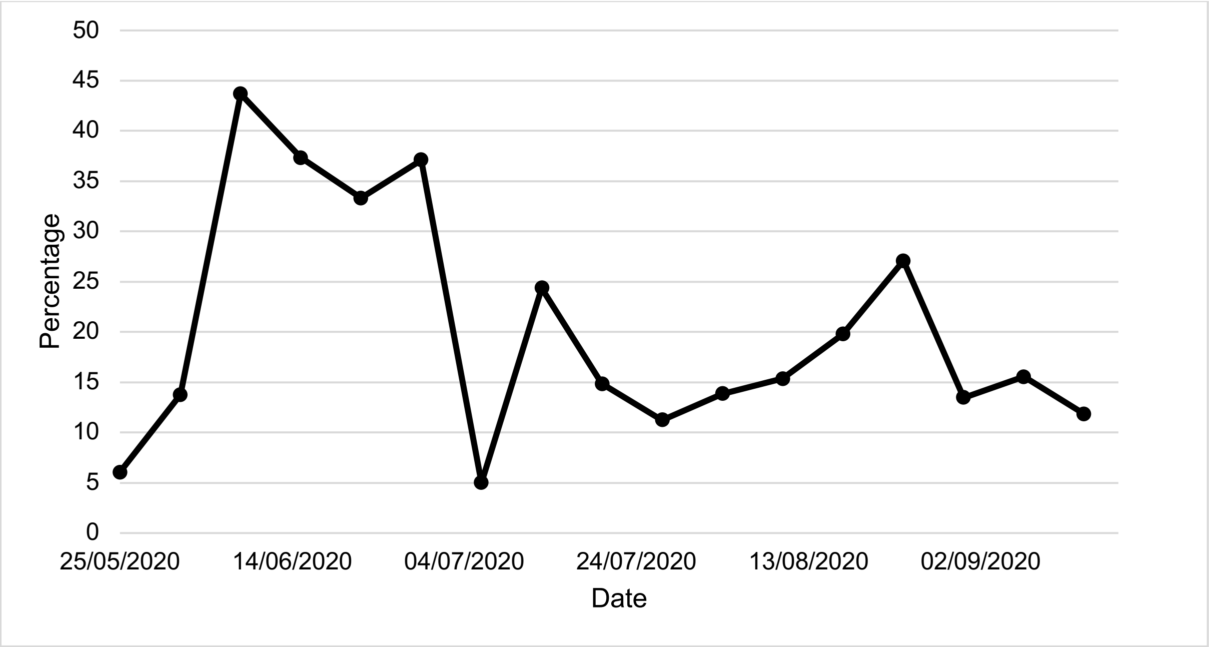

Supplementary Figure 14: Percentage of known SARS-CoV-2 cases sequenced in England from 25/05/2020 to 14/09/2020

Supplementary Tables

Study	Month (2020)	Country	Imported from	No. imports	Genomes
Au et al	March	China	US (2), Germany (1)	3	7
Bohmer et al	January	Germany	China	1	
Cohen-Gihon et al		Israel	Japan (1), Italy (1)	2	
Jesus et al		Brazil	Italy (4)		6
Nascimento et al	March	Brazil	Spain	1	
Du et al	Before May	China	South America (2), North America (15), Europe (101), Other Asian countries (3)		102
Garces-Ayala et al	February	Mexico	Italy	1	
Giandhari et al	February - March	South Africa	Europe	13	27
Giovanetti et al	January	Italy	China	2	
Gomez-Carbella et al	Before June	Spain		> 34	
Gong et al	January - March	Taiwan	China, Germany, UK, Turkey, Iran, Middle East, Europe		20
Jia et al	March	China	Spain (2), France (1), Cambodia (1), Sri Lanka (1), US (3)	8	
Kouriba et al	April	Mali			21
Kumar et al		India	China, South Asia, Middle East, Italy, Spain, UK, France and USA		104
Liu et al	April	China	US	1	
Lu et al	January - March	China	19 different countries	102	53 new + 177 publicly available sequences
Manning et al	January	Cambodia		1	
Marquez et al	March	Ecuador	Netherlands	1	4
Puenpa et al	<May	Thailand	China	1	40
Rockett et al	January - March	Australia	Asia, Western Europe and North America		
Seemann et al		Australia	Asia, Europe, North America	193	76 clusters, 34 only international travel, 34 mixed local/international
Sekizuka et al	March	Japan	Egypt	10	26
Stange et al	February - March	Switzerland	Italy (2), France (1), Austria (refers to a previous study)		486
Tapfumani et al	February - March	Zimbabwe	UK, US, South Africa, Dubai		97

Supplementary Table 1: Studies using genomics to as part of epidemiological investigations of importations of SARS-CoV-2.

Ethnic group	Cases		Census
**White**	**No.**	**%**	%
English/Welsh/Scottish/Northern Irish/British	2509	66.8%	80.5%
Irish	35	0.9%	0.9%
Gypsy or Irish Traveller	0	0.0%	0.1%
Any other White background	583	15.5%	4.4%
**Mixed/Multiple ethnic groups**			
White and Black Caribbean	42	1.1%	0.8%
White and Black African	25	0.7%	0.3%
White and Asian	49	1.3%	0.6%
Any other Mixed/Multiple ethnic background	44	1.2%	0.5%
**Asian/Asian British**			
Indian	103	2.7%	2.5%
Pakistani	79	2.1%	2.0%
Bangladeshi	27	0.7%	0.8%
Chinese	6	0.2%	0.7%
Any other Asian background	62	1.7%	1.5%
**Black/ African/Caribbean/Black British**			
African	58	1.5%	1.8%
Caribbean	12	0.3%	1.1%
Any other Black/African/Caribbean background	12	0.3%	0.5%
**Other ethnic group**			
Arab	0	0.0%	0.4%
Any other ethnic group	110	2.9%	0.6%
**Other**			
Prefer not to say	65		
Unknown	386		

Supplementary Table 2: Self-identified ethnicity of cases (UK Government Statistical Service ethnic groups). The 2011 census data for England and Wales was used.

Demographic	Cases		Contacts		Cases with Genomes that passed QC	
**Sex**						
Male	2193	56.2%	6088	49.7%	394	47.8%
Female	1933	46.8%	6160	50.3%	414	50.2%
Unknown	81		6607		19	
**Age**						
0-5	51	1.2%	303	2.5%	9	1.1%
6-10	45	1.1%	361	2.9%	12	1.5%

11-15	75	1.8%	467	3.8%	14	1.7%
16-20	1086	25.8%	2274	18.5%	228	27.6%
21-25	843	20.0%	1866	15.2%	165	20.0%
26-30	685	16.3%	1350	11.0%	135	16.3%
31-35	413	9.8%	849	6.9%	88	10.6%
36-40	278	6.6%	721	5.9%	46	5.6%
41-45	185	4.4%	691	5.6%	35	4.2%
46-50	169	4.0%	984	8.0%	34	4.1%
51-55	130	3.1%	1168	9.5%	22	2.7%
56-60	121	2.9%	692	5.6%	23	2.8%
61-65	57	1.4%	264	2.1%	7	0.8%
66-70	30	0.7%	141	1.1%	4	0.5%
71-75	20	0.5%	101	0.8%	4	0.5%
76-80	7	0.2%	35	0.3%	0	0.0%
81-85	7	0.2%	30	0.2%	1	0.1%
86-90	3	0.1%	7	0.1%	0	0.0%
91-95	0	0.0%	4	0.0%	0	0.0%
Unknown	2		6547		0	
**Ethnic group**						
**White**						
English/Welsh/Scottish/Northern Irish/British	2509	66.8%	8370	73.8%	499	68.4%
Irish	35	0.9%	134	1.2%	4	0.5%
Gypsy or Irish Traveller	0	0.0%	0	0.0%	0	0.0%
Any other White background	583	15.5%	1261	11.1%	120	16.4%
**Mixed/Multiple ethnic groups**						
White and Black Caribbean	42	1.1%	88	0.8%	4	0.5%
White and Black African	25	0.7%	60	0.5%	8	1.1%
White and Asian	49	1.3%	112	1.0%	9	1.2%
Any other Mixed/Multiple ethnic background	44	1.2%	124	1.1%	9	1.2%
**Asian/Asian British**						
Indian	103	2.7%	318	2.8%	13	1.8%
Pakistani	79	2.1%	183	1.6%	4	0.5%
Bangladeshi	27	0.7%	50	0.4%	3	0.4%
Chinese	6	0.2%	33	0.3%	1	0.1%
Any other Asian background	62	1.7%	197	1.7%	13	1.8%
**Black/ African/Caribbean/Black British**						
African	58	1.5%	148	1.3%	12	1.6%
Caribbean	12	0.3%	35	0.3%	3	0.4%
Any other Black/African/Caribbean background	12	0.3%	25	0.2%	2	0.3%
**Other ethnic group**						

Arab	0	0.0	0	0.0%	0	0.0%
Any other ethnic group	110	2.9%	195	1.7%	26	3.6%
**Other**						
Prefer not to say	65		139		85	
Unknown	386		7382		12	
**Region**						
London	1205	28.6%	4681	24.9%	298	36.3%
South East	623	14.8%	3201	17.0%	175	21.3%
North West	584	13.9%	2503	13.3%	70	8.5%
East of England	395	9.4%	1958	10.4%	94	11.4%
South West	328	7.8%	1755	9.3%	74	9.0%
Yorkshire and Humber	327	7.8%	1488	7.9%	21	2.6%
West Midlands	299	7.1%	1410	7.5%	20	2.4%
East Midlands	251	6.0%	1157	6.2%	29	3.5%
North East	161	3.8%	646	3.4%	40	4.9%
Not stated	34	0.8%	56		6	

Supplementary Table 3: Demographics of cases, contacts, and cases with genomes that pass quality control available

Country	Cases		Sequenced samples from cases (passed QC)		Percentage of cases sequenced by country of travel
	N	%	N	%	18.8%
Greece	882	21.0%	166	20.1%	23.6%
Croatia	685	16.3%	162	19.6%	18.0%
Spain	589	14.0%	106	12.8%	20.2%
Unknown	282	6.7%	57	6.9%	23.3%
France	223	5.3%	52	6.3%	11.2%
Turkey	187	4.4%	21	2.5%	18.0%
Portugal	111	2.6%	20	2.4%	15.2%
Malta	99	2.4%	15	1.8%	22.6%
Italy	93	2.2%	21	2.5%	16.5%
Poland	85	2.0%	14	1.7%	16.7%
Romania	78	1.9%	13	1.6%	15.4%
Czech Republic	65	1.5%	10	1.2%	19.7%
Albania	61	1.4%	12	1.5%	27.9%
Hungary	61	1.4%	17	2.1%	24.6%
India	57	1.4%	14	1.7%	21.8%
Pakistan	55	1.3%	12	1.5%	13.2%
Netherlands	38	0.9%	5	0.6%	20.7%
Germany	29	0.7%	6	0.7%	25.0%
Switzerland	28	0.7%	7	0.8%	12.5%
Kosovo	24	0.6%	3	0.4%	18.8%

Total cases	4207		827		19.7%

Supplementary Table 4: The top 20 countries reported as the travel destination for importations of SARS-CoV-2 into England and the associated number of samples sequenced from travel-related cases

Demographic	Cases	Total contacts of cases	contacts reported per case
**Sex**			
Male	2193	9835	4.5
Female	1933	8578	4.4
Unknown	82	224	3.1
**Age**			
0-5	51	183	3.6
6-10	45	124	2.8
11-15	75	321	4.3
16-20	1086	7473	6.9
21-25	843	3536	4.2
26-30	685	2091	3.1
31-35	413	1312	3.2
36-40	278	939	3.4
41-45	185	566	3.1
46-50	169	723	4.3
51-55	130	469	3.6
56-60	121	434	3.6
61-65	57	229	4.0
66-70	30	116	3.9
71-75	20	63	3.2
76-80	7	16	2.3
81-85	7	39	5.6
86-90	3	3	1.0
91-95	0	0	NA
Unknown	2		
**Ethnic group**			
**White**			
English/Welsh/Scottish/Northern Irish/British	2509	12745	5.1
Irish	35	121	3.5
Gypsy or Irish Traveller	0	0	NA
Any other White background	583	1755	3.0
**Mixed/Multiple ethnic groups**			
White and Black Caribbean	42	284	6.8
White and Black African	25	108	4.3

White and Asian	49	216	4.4
Any other Mixed/Multiple ethnic background	44	147	3.3
**Asian/Asian British**			
Indian	103	481	4.7
Pakistani	79	306	3.9
Bangladeshi	27	82	3.0
Chinese	6	2	0.3
Any other Asian background	62	199	3.2
**Black/ African/Caribbean/Black British**			
African	58	152	2.6
Caribbean	12	36	3.0
Any other Black/African/Caribbean background	12	28	2.3
**Other ethnic group**			
Arab	0	0	NA
Any other ethnic group	110	367	3.3
**Other**			
Prefer not to say	65	135	2.1
Unknown	386	1473	3.8
**Region**			
London	1205	4275	3.5
South East	622	3211	5.2
North West	584	2323	4.0
East of England	395	2079	5.3
South West	328	1960	6.0
Yorkshire and Humber	327	1411	4.3
West Midlands	299	1259	4.2
East Midlands	251	1351	5.4
North East	161	660	4.1
Not stated	35	108	3.1
**Country**			
Greece	882	5587	6.3
Croatia	685	3913	5.7
Spain	589	1521	2.6
Unknown	282	988	3.5
France	223	815	3.7
Turkey	187	702	3.8
Portugal	111	439	4.0
Malta	99	492	5.0

Italy	93	390	4.2
Poland	85	417	4.9
Romania	78	189	2.4
Hungary	67	137	2.0
Czech Republic	66	239	3.6
Albania	61	140	2.3
India	57	223	3.9
Pakistan	55	269	4.9
Netherlands	38	166	4.4
Germany	29	101	3.5
Switzerland	28	123	4.4
Kosovo	24	64	2.7

Supplementary Table 5: Contacts per case related to Sex, Age, Ethnic Group, Region of residence and reported Travel Destination

	Effect of travel restriction (ratio of mean contacts)		Adjusted mean contacts	
	**Unadjusted rate ratio**	**Adjusted rate ratio**	**With travel restriction**	**Without travel restriction**
**Overall**	0.50 (0.47-0.54)	0.60 (0.37-0.95)	3.50 (3.04-4.02)	5.85 (3.67-9.34)
**By age-group**				
0-15	0.75 (0.50-1.12)	0.73 (0.39-1.34)	4.3 (3.3-5.6)	5.9 (3.3-10.3)
16-20	0.50 (0.42-0.59)	0.52 (0.32-0.85)	4.7 (3.9-5.7)	9.0 (5.6-14.5)
21-25	0.51 (0.43-0.61)	0.54 (0.33-0.88)	3.5 (2.9-4.2)	6.5 (4.0-10.5)
26-30	0.47 (0.38-0.56)	0.49 (0.30-0.80)	2.4 (2.0-2.9)	4.8 (3.0-7.8)
31-40	0.62 (0.51-0.75)	0.58 (0.36-0.94)	3.0 (2.5-3.6)	5.2 (3.2-8.3)
41 and older	0.84 (0.69-1.03)	0.78 (0.48-1.29)	3.7 (3.1-4.3)	4.7 (2.9-7.6)
**By calendar date**				
May/June			5.9 (4.3-8.2)	Insufficient data
July	0.71 (0.50-1.01)	0.72 (0.51-1.03)	5.1 (4.0-6.4)	7.0 (5.2-9.5)
August 1-14	0.30 (0.24-0.37)	0.42 (0.33-0.53)	2.5 (2.1-3.1)	6.1 (5.0-7.4)
August 15-31	0.45 (0.41-0.50)	0.60 (0.52-0.69)	2.5 (2.1-2.8)	4.1 (3.5-4.8)
September	0.66 (0.57-0.77)	0.78 (0.65-0.93)	2.8 (2.4-3.2)	3.6 (3.0-4.2)

Supplementary Table 6: The effect of travel restriction (14 day quarantine) on reported contacts per imported case, and the estimated marginal mean number of reported cases when imported from a country with or without a travel restriction in place. Figures are reported for the overall dataset, and then stratified by age-group and calendar date of positive test. All figures are estimated marginal means or marginal effects provided with 95% confidence intervals.

Lineage	No. Samples	Percentage
UK5	152	18.4%
UK1897	73	8.8%

UK461	66	8.0%
UK2229	28	3.4%
UK1249	22	2.7%
UK649	22	2.7%
UK1506	22	2.7%
UK1031	12	1.5%
UK1205	10	1.2%
UK2347	10	1.2%
UK761	10	1.2%
UK1780	8	1.0%
UK1791	8	1.0%
UK1569	7	0.8%
UK831	7	0.8%
UK669	7	0.8%
UK1018	6	0.7%
UK1219	6	0.7%
UK2683	6	0.7%
UK2726	6	0.7%
UK778	5	0.6%
UK1535	5	0.6%
UK1581	5	0.6%
UK2268	5	0.6%
214 lineages with <5 cases	319	38.6%

Supplementary Table 7: The number of samples with each UK lineage

**Lineage**	**No. Samples**	**Percentage**
B.1.1	159	19.2%
B.1.177	128	15.5%
D.1	87	10.5%

B.1.160	75	9.1%
B.1.5	72	8.7%
B.1	72	8.7%
B.1.1.1	55	6.7%
B.1.1.37	55	6.7%
B.1.78	36	4.4%
B.1.1.70	17	2.1%
B.1.36	14	1.7%
B.1.5.12	6	0.7%
B.1.36.1	6	0.7%
B.1.1.34	5	0.6%
25 lineages with <5 cases	40	4.8%

Supplementary Table 8: The number of travel-related samples with each Global lineage

Lineage	Number	Percentage
B.1.1	7673	37.2
B.1.177	1862	9.0
B.1	1547	7.5
B.1.5	1299	6.3
B.1.1.37	1173	5.7
B.1.1.35	996	4.8
B.1.1.1	805	3.9
D.1	647	3.1
B.1.160	405	2.0
B.1.36.1	362	1.8
B.1.1.4	335	1.6
B	321	1.6
B.1.1.51	319	1.5
B.1.1.30	233	1.1
B.1.36	232	1.1
B.1.1.15	189	0.9
B.1.78	181	0.9
B.1.1.55	145	0.7
C.3	125	0.6
B.1.1.70	112	0.5

Supplementary Table 9: The number of samples with each Global lineage from the COG-UK dataset during the study period. This table includes the ‘top 20’ lineages sequenced during the study period.

Mutation	Cases with Mutant Variant		Wild Type Variant		Inconclusive	
D614G	824	99.64%	3	0.36%	0	0.00%
P323L*	4	0.48%	815	98.55%	7	0.85%

N439K	65	7.86%	758	91.66%	4	0.48%
A222V	131	15.84%	694	83.92%	2	0.24%
Y453F	0	0.00%	826	99.88%	1	0.12%
Total Cases						1114

Supplementary Table 10: Mutant variants identified in the travel-related cases during the study period. Letters in the amino acid substitution nomenclature correspond to: A, alanine; D, aspartic acid; F, phenylalanine; G, glycine; K, lysine; L, leucine; N, asparagine; P, proline; V, valine; Y, tyrosine. The mutations are named as following: the letter preceding number (the amino acid site of substitution) represents the wild-type amino acid, the letter following the number is the observed amino acid in the sample (‘a mutation’, if different from the wild-type). *F mutation found in 1 case.

Virus name	Accession ID	Originating Laboratory	Submitting Laboratory	Authors
**hCoV-19/Switzerland/ZH-ETHZ-260011/2020**	EPI_ISL_539348	Viollier AG	Department of Biosystems Science and Engineering, ETH Zürich	Christian Beisel; Christiane Beckmann; Christoph Noppen; Elodie Burcklen; Ina Nissen; Ivan Topolsky; Maurice Redondo; Natascha Santacroce; Niko Beerenwinkel; Noemie Santamaria de Souza; Olivier Kobel; Pedro Ferreira; Philipp Jablonski; Sarah Nadeau; Sophie Seidel; Susana Posada-Céspedes; Tanja Stadler; Tobias Schär
**hCoV-19/Germany/NW-HHU-90/2020**	EPI_ISL_539606	ZOTZ KLIMAS MVZ Düsseldorf-Centrum GbR ÜBAG für Labormedizin, Genetik, Zytologie, Pathologie	Center of Medical Microbiology, Virology, and Hospital Hygiene, University of Duesseldorf	Alexander Dilthey; Andreas Walker; Ashley-Jane Duplessis; Daniel Strelow; Jessica Nicolai; Jörg Timm; Katrin Hoffmann; Klaus Pfeffer; Malte Kohns Vasconcelos; Marek Korencak; Maximilian Damagnez; Nadine Lübke; Patrick Finzer; Rainer Zotz; Tobias Wienemann; Torsten Houwaart
**hCoV-19/Switzerland/AG-ETHZ-260057/2020**	EPI_ISL_539383	Viollier AG	Department of Biosystems Science and Engineering, ETH Zürich	Christian Beisel; Christiane Beckmann; Christoph Noppen; Elodie Burcklen; Ina Nissen; Ivan Topolsky; Maurice Redondo; Natascha Santacroce; Niko Beerenwinkel; Noemie Santamaria de Souza; Olivier Kobel; Pedro Ferreira; Philipp Jablonski; Sarah Nadeau; Sophie Seidel; Susana Posada-Céspedes; Tanja Stadler; Tobias Schär
**hCoV-19/Switzerland/AG-ETHZ-260154/2020**	EPI_ISL_539449	Viollier AG	Department of Biosystems Science and Engineering, ETH Zürich	Christian Beisel; Christiane Beckmann; Christoph Noppen; Elodie Burcklen; Ina Nissen; Ivan Topolsky; Maurice Redondo; Natascha Santacroce; Niko Beerenwinkel; Noemie Santamaria de Souza; Olivier Kobel; Pedro Ferreira; Philipp Jablonski; Sarah Nadeau; Sophie Seidel; Susana Posada-Céspedes; Tanja Stadler; Tobias Schär
**hCoV-19/Switzerland/BE-ETHZ-260043/2020**	EPI_ISL_539373	Viollier AG	Department of Biosystems Science and Engineering, ETH Zürich	Christian Beisel; Christiane Beckmann; Christoph Noppen; Elodie Burcklen; Ina Nissen; Ivan Topolsky; Maurice Redondo; Natascha Santacroce; Niko Beerenwinkel; Noemie Santamaria de Souza; Olivier Kobel; Pedro Ferreira; Philipp Jablonski; Sarah Nadeau; Sophie Seidel; Susana Posada-Céspedes; Tanja Stadler; Tobias Schär
**hCoV-19/Switzerland/BE-ETHZ-260152/2020**	EPI_ISL_539447	Viollier AG	Department of Biosystems Science and Engineering, ETH Zürich	Christian Beisel; Christiane Beckmann; Christoph Noppen; Elodie Burcklen; Ina Nissen; Ivan Topolsky; Maurice Redondo; Natascha Santacroce; Niko Beerenwinkel; Noemie Santamaria de Souza; Olivier Kobel; Pedro Ferreira; Philipp Jablonski; Sarah Nadeau; Sophie Seidel; Susana Posada-Céspedes; Tanja Stadler; Tobias Schär
**hCoV-19/Switzerland/VD-ETHZ-260040/2020**	EPI_ISL_539370	Viollier AG	Department of Biosystems Science and Engineering, ETH Zürich	Christian Beisel; Christiane Beckmann; Christoph Noppen; Elodie Burcklen; Ina Nissen; Ivan Topolsky; Maurice Redondo; Natascha Santacroce; Niko Beerenwinkel; Noemie Santamaria de Souza; Olivier Kobel; Pedro Ferreira; Philipp Jablonski; Sarah Nadeau; Sophie Seidel; Susana Posada-Céspedes; Tanja Stadler; Tobias Schär

**hCoV-19/Switzerland/VD-ETHZ-260053/2020**	EPI_ISL_539380	Viollier AG	Department of Biosystems Science and Engineering, ETH Zürich	Christian Beisel; Christiane Beckmann; Christoph Noppen; Elodie Burcklen; Ina Nissen; Ivan Topolsky; Maurice Redondo; Natascha Santacroce; Niko Beerenwinkel; Noemie Santamaria de Souza; Olivier Kobel; Pedro Ferreira; Philipp Jablonski; Sarah Nadeau; Sophie Seidel; Susana Posada-Céspedes; Tanja Stadler; Tobias Schär
**hCoV-19/Switzerland/VD-ETHZ-260076/2020**	EPI_ISL_539398	Viollier AG	Department of Biosystems Science and Engineering, ETH Zürich	Christian Beisel; Christiane Beckmann; Christoph Noppen; Elodie Burcklen; Ina Nissen; Ivan Topolsky; Maurice Redondo; Natascha Santacroce; Niko Beerenwinkel; Noemie Santamaria de Souza; Olivier Kobel; Pedro Ferreira; Philipp Jablonski; Sarah Nadeau; Sophie Seidel; Susana Posada-Céspedes; Tanja Stadler; Tobias Schär
**hCoV-19/Switzerland/VD-ETHZ-260077/2020**	EPI_ISL_539399	Viollier AG	Department of Biosystems Science and Engineering, ETH Zürich	Christian Beisel; Christiane Beckmann; Christoph Noppen; Elodie Burcklen; Ina Nissen; Ivan Topolsky; Maurice Redondo; Natascha Santacroce; Niko Beerenwinkel; Noemie Santamaria de Souza; Olivier Kobel; Pedro Ferreira; Philipp Jablonski; Sarah Nadeau; Sophie Seidel; Susana Posada-Céspedes; Tanja Stadler; Tobias Schär
**hCoV-19/Switzerland/VD-ETHZ-260090/2020**	EPI_ISL_539411	Viollier AG	Department of Biosystems Science and Engineering, ETH Zürich	Christian Beisel; Christiane Beckmann; Christoph Noppen; Elodie Burcklen; Ina Nissen; Ivan Topolsky; Maurice Redondo; Natascha Santacroce; Niko Beerenwinkel; Noemie Santamaria de Souza; Olivier Kobel; Pedro Ferreira; Philipp Jablonski; Sarah Nadeau; Sophie Seidel; Susana Posada-Céspedes; Tanja Stadler; Tobias Schär
**hCoV-19/Switzerland/VD-ETHZ-260145/2020**	EPI_ISL_539441	Viollier AG	Department of Biosystems Science and Engineering, ETH Zürich	Christian Beisel; Christiane Beckmann; Christoph Noppen; Elodie Burcklen; Ina Nissen; Ivan Topolsky; Maurice Redondo; Natascha Santacroce; Niko Beerenwinkel; Noemie Santamaria de Souza; Olivier Kobel; Pedro Ferreira; Philipp Jablonski; Sarah Nadeau; Sophie Seidel; Susana Posada-Céspedes; Tanja Stadler; Tobias Schär
**hCoV-19/Switzerland/VD-ETHZ-260177/2020**	EPI_ISL_539468	Viollier AG	Department of Biosystems Science and Engineering, ETH Zürich	Christian Beisel; Christiane Beckmann; Christoph Noppen; Elodie Burcklen; Ina Nissen; Ivan Topolsky; Maurice Redondo; Natascha Santacroce; Niko Beerenwinkel; Noemie Santamaria de Souza; Olivier Kobel; Pedro Ferreira; Philipp Jablonski; Sarah Nadeau; Sophie Seidel; Susana Posada-Céspedes; Tanja Stadler; Tobias Schär
**hCoV-19/Switzerland/VS-ETHZ-260042/2020**	EPI_ISL_539372	Viollier AG	Department of Biosystems Science and Engineering, ETH Zürich	Christian Beisel; Christiane Beckmann; Christoph Noppen; Elodie Burcklen; Ina Nissen; Ivan Topolsky; Maurice Redondo; Natascha Santacroce; Niko Beerenwinkel; Noemie Santamaria de Souza; Olivier Kobel; Pedro Ferreira; Philipp Jablonski; Sarah Nadeau; Sophie Seidel; Susana Posada-Céspedes; Tanja Stadler; Tobias Schär

Supplementary Table 11: GISAID acknowledgement table. We gratefully acknowledge the Authors in Supplementary Table 11 from the Originating laboratories responsible for obtaining the specimens, as well as the Submitting laboratories where the genome data were generated and shared via GISAID[2], on which this research is based. All Submitters of data may be contacted directly via www.gisaid.org. Authors are sorted alphabetically.

Supplementary Methods

Travel guidance
During the time period of the study all non-essential travel outside of the UK was advised against. Varying restrictions were applied to travellers returning from different countries or regions of countries, changing over the course of the study period. Travellers were required to quarantine for 2 weeks if they had visited a restricted region in the previous 2 weeks. There were exceptions for particular classes of individuals such as freight drivers and flight crews. Designated regions were exempt from the quarantine requirements, and commonly referred to as open 'travel-corridors'. These restrictions changed over time for different regions (https://www.gov.uk/guidance/travel-advice-novel-coronavirus).

Contact tracing and case identification
Contact-tracing data was obtained from T&T. Case data gathered from testing laboratories is enriched with data provided by NHS Spine, prior to arrival at the contact tracing advise service system. All cases and contacts had a field for demographic data, but this was not always reported (Table 2 and Supplementary Table 3). 'Highly probable' travel-related cases were defined as individuals who reported international travel as an activity in the two days before symptom onset/testing. On 12/08/2020 the additional facility to report international travel in the seven days prior to symptom onset/testing became available, and also included in this study and defined as 'probable' travel-related cases.

Cases are asked to provide details of all contacts for activities in the 2 days prior to onset/testing up to completing the system which were gathered. If any contacts become cases they would then also be included in T&T as a case separately but if they did not report direct travel themselves, then they would not meet the definition for a travel-associated case. Positive cases are contacted by Test and Trace via online or call centre tracing. Additionally travel-related cases are seen as higher risk and therefore referred to local public health agencies for targeted contact tracing

Cases identified reporting travel in 7-day period prior to symptom onset or positive test:
Test and Trace data included destination city, and a free-text search was run with a custom python script to convert city to associated country of destination. All fields were manually cross-checked and any errors corrected (142 corrections). A further 103 countries were manually inputted due to spelling errors in the free-text Test and Trace data provided and 1 country by searching flight numbers provided by the case when country or city not available. 22 cases reporting travel-related activities did not have an associated destination clearly identified.

Cases identified reporting travel in 2-day period prior to symptom onset or positive test:
A free-text country and city search with a custom python script on travel-related T&T data was used to identify destination country. This yielded 1898 destination countries, and a further 1182 by city search. All fields were manually cross-checked and errors corrected (210 corrected). 542 case-country associations were manually entered where spelling mistakes were present in the free-text entries, including 98 entered by flight number searches where this was the only available data.

Lineages
Global and UK Lineages[3] were assigned to each genome using Pangolin (https://github.com/cov-lineages/pangolin) with analysis performed on COVID-CLIMB[4]. Global lineages, reflecting genomically distinct identifiable importations into a new region, are denoted with a letter followed by a hierarchy of up to 4 numbers such as B.1.2.3, providing for a stable and consistent naming of clusters. These lineages are manually curated and assigned. UK lineages represent the subsequent regional and local spread within the UK, taking the form UK1234, providing an identifier for a cluster for a given phylogeny. These identifiers are assigned programmatically are unstable. Labelled phylogenetic trees were created using CIVET tool (version 2.0) (https://github.com/cog-uk/civet).

Identification of extinct and unique genomes
The 827 high-quality travel-related genomes were compared to the COG-UK dataset on 16/10/2020. Genomes were only compared to other genomes with the same UK lineage assigned by COG-UK, since we assume that no relatedness relevant to transmission exists between genomes of different UK lineages. A unique genome in the community was deemed to be one that was known to be from a

travel-related case and either: (1) A UK lineage that had not been sampled in the previous 4 weeks in the UK, (2) >3 SNPs distance to the closest relative in the COG-UK dataset.
Within the same UK lineage we identified those genomes sampled within 4 weeks prior to the genome of interest. We determined the minimum SNP distance between the sequence of interest and these genomes. This identified 207/827 genomes with a minimum SNP distance of >3 SNPs to its closest relative in the COG-UK dataset. These constitute genomes for which no close relative was sampled in the UK at the time of importation. The analysis was then repeated on 05/12/2020 on these 207 'unique genomes' to account for delays in genomes uploaded to MRC CLIMB. 195/207 were included in this analysis, with 12/207 genomes excluded due to the large UK phylotypes they belonged to and the subsequent computational requirements. At this time a further 8 genomes were determined to have a close relative sampled in the UK in the 4 weeks before importation. This wasn't detected earlier, because their close relative was uploaded with a significant delay.
The remaining 186 genomes were 'Unique' genomes were compared to sequences that were generated in the COG-UK dataset within 2 and 4 weeks after their sampling date, to identify samples with the same UK lineage and within 2 SNPs. These would represent onward transmission or further introductions of similar genomes. The analysis was run with an in-house custom Python script developed by US and RM.

Supplementary References

1 Care, D. f. H. a. S. *Weekly statistics for NHS Test and Trace (England)*, <https://www.gov.uk/government/collections/nhs-test-and-trace-statistics-england-weekly-reports> (2020).

2 Shu, Y. & McCauley, J. GISAID: Global initiative on sharing all influenza data - from vision to reality. *Euro Surveill* **22**, doi:10.2807/1560-7917.ES.2017.22.13.30494 (2017).

3 Rambaut, A. *et al.* A dynamic nomenclature proposal for SARS-CoV-2 lineages to assist genomic epidemiology. *Nat Microbiol* **5**, 1403-1407, doi:10.1038/s41564-020-0770-5 (2020).

4 Connor, T. R. *et al.* CLIMB (the Cloud Infrastructure for Microbial Bioinformatics): an online resource for the medical microbiology community. *Microb Genom* **2**, e000086, doi:10.1099/mgen.0.000086 (2016).
