# Supplementary material for: Genomic assessment of quarantine measures to prevent SARS-CoV-2 importation and transmission
Source: Nat Commun. 2022 Feb 23;13:1012. doi: 10.1038/s41467-022-28371-z (PMC8866425; doi:10.1038/s41467-022-28371-z)
Supplement: Supplementary file 3 — Description of Additional Supplementary Files [file 41467_2022_28371_MOESM3_ESM.pdf]

Description of Additional Supplementary Files

File Name: Supplementary Data 1
Description: ENA accession codes of imported sequences used in this study
