# Supplementary material for: Genomic assessment of quarantine measures to prevent SARS-CoV-2 importation and transmission
Source: Nat Commun. 2022 Feb 23;13:1012. doi: 10.1038/s41467-022-28371-z (PMC8866425; doi:10.1038/s41467-022-28371-z)
Supplement: Supplementary file 5 — Reporting Summary [file 41467_2022_28371_MOESM5_ESM.pdf]

nature research

Corresponding author(s): Dinesh Aggarwal, Ewan Harrison

Last updated by author(s): Dec 2, 2021

Reporting Summary

Nature Research wishes to improve the reproducibility of the work that we publish. This form provides structure for consistency and transparency in reporting. For further information on Nature Research policies, see our Editorial Policies and the Editorial Policy Checklist.

Statistics

For all statistical analyses, confirm that the following items are present in the figure legend, table legend, main text, or Methods section.

n/a	Confirmed	
☐	☒	The exact sample size (*n*) for each experimental group/condition, given as a discrete number and unit of measurement
☒	☐	A statement on whether measurements were taken from distinct samples or whether the same sample was measured repeatedly
☐	☒	The statistical test(s) used AND whether they are one- or two-sided<br>*Only common tests should be described solely by name; describe more complex techniques in the Methods section.*
☐	☒	A description of all covariates tested
☐	☒	A description of any assumptions or corrections, such as tests of normality and adjustment for multiple comparisons
☐	☒	A full description of the statistical parameters including central tendency (e.g. means) or other basic estimates (e.g. regression coefficient) AND variation (e.g. standard deviation) or associated estimates of uncertainty (e.g. confidence intervals)
☐	☒	For null hypothesis testing, the test statistic (e.g. *F*, *t*, *r*) with confidence intervals, effect sizes, degrees of freedom and *P* value noted<br>*Give P values as exact values whenever suitable.*
☒	☐	For Bayesian analysis, information on the choice of priors and Markov chain Monte Carlo settings
☒	☐	For hierarchical and complex designs, identification of the appropriate level for tests and full reporting of outcomes
☒	☐	Estimates of effect sizes (e.g. Cohen's *d*, Pearson's *r*), indicating how they were calculated

Our web collection on statistics for biologists contains articles on many of the points above.

Software and code

Policy information about availability of computer code

Data collection	Destination countries for cases were originally identified from freetext entries using a custom python script written by author, SP, which can be made available. Data was then manually checked to ensure accuracy.
Data analysis	Sequences were assembled using the ARTIC pipeline (Nanopolish) v1.0.0 (https://github.com/artic-network/fieldbioinformatics). Genomes were aligned with MAFFT (version7.471). Extinct and unique genomes were identified using a custom python script developed by authors US and RM, available at https://github.com/COG-UK/travel-quarantine/. Global and UK Lineages (Rambaut et al. 2020) were assigned to each genome using Pangolin (https://github.com/cov-lineages/pangolin/releases/tag/v2.0.8) with analysis performed on MRC CLIMB (Connor et al. 2016). Minor variants were pre-defined within the COG-UK database using type_variants (https://github.com/cov-ert/type_variants) (no version release). SNP differences were calculated with snp-dists (https://github.com/tseemann/snp-dists/releases/tag/v0.7.0). All models were estimated using the glmmTMB package (version 1.0.1) (Brooks et al. 2017) with marginal means and effects calculated using the emmeans package (1.5.2-1) (Lenth et al. 2018) for R (version 3.5.1) (Team 2018). Figures were generated with RStudio (version 1.3.1093) using R (version 4.0.2) and Microsoft Excel (version 1908). We used the Polecat clustering tool (https://cog-uk.github.io/polecat) (no version release) to systematically identify outliers in COG-UK genomic dataset.<br><br>Custom code used in this analysis is available at https://github.com/COG-UK/travel-quarantine/. Please direct further queries to the corresponding authors.

For manuscripts utilizing custom algorithms or software that are central to the research but not yet described in published literature, software must be made available to editors and reviewers. We strongly encourage code deposition in a community repository (e.g. GitHub). See the Nature Research guidelines for submitting code & software for further information.

Data

Policy information about availability of data

All manuscripts must include a data availability statement. This statement should provide the following information, where applicable:

- Accession codes, unique identifiers, or web links for publicly available datasets
- A list of figures that have associated raw data
- A description of any restrictions on data availability

Assembled/consensus genomes are available from GISAID subject to minimum quality control criteria. Raw reads are available from European Nucleotide Archive (ENA) under accession PRJEB37886. ENA accession codes for travel-related genomes used in this study are available in supplementary materials (Supplementary Data 1) and available from GitHub at https://github.com/COG-UK/travel-quarantine/. The Genbank accession code for the reference genome uses is MN908947.3. All genomes, phylogenetic trees, basic metadata are available from the COG-UK consortium website (https://www.cogconsortium.uk/data). Extensive aggregated metadata has been made available in supplementary files. Genomes accessed through GISAID used in this study are provided in the Supplementary Information file entitled 'GISAID acknowledgment table'. For confidentiality reasons, extended metadata are under restricted access; requests for access should be directed to corresponding authors and specifically for Public Health England data, to the Public Health England office of data release (https://www.gov.uk/government/publications/accessing-public-health-england-data/about-the-phe-odr-and-accessing-data) with an estimated 60 working days turnaround time.

Field-specific reporting

Please select the one below that is the best fit for your research. If you are not sure, read the appropriate sections before making your selection.

- [x] Life sciences
- [ ] Behavioural & social sciences
- [ ] Ecological, evolutionary & environmental sciences

For a reference copy of the document with all sections, see nature.com/documents/nr-reporting-summary-flat.pdf

Life sciences study design

All studies must disclose on these points even when the disclosure is negative.

Sample size	The study is an epidemiological study that is observational in nature. The study involves the analysis of data from all available imported cases of SARS-CoV-2 and if available, their genomes. Given it is a descriptive study based on surveillance data, there were no sample size calculations conducted.
Data exclusions	The vast majority of available data was included in the study. Duplicate cases were excluded. Sequences with higher ambiguous site content (>10%) were excluded as they can result in false inferences of genomic clustering and inaccuracies in analysis.
Replication	We point out this is a descriptive epidemiological study with no experiments being conducted for replication. We have successfully repeated the analysis aimed to identify unique genomes as new data became available, conducted by 2 independent researchers (DA and US). All sequencing data is publicly available to enable other researchers to replicate findings.
Randomization	This study reports genomic epidemiology derived from prospectively collected surveillance data. It describes the impact of a non-pharmacological intervention (travel-related quarantine) implemented at a national level through governmental guidelines with individuals included in the study required to follow guidance through law and therefore randomization is not appropriate.
Blinding	Blinding is not applicable to this study as access to epidemiological and contact tracing data was required identify the requirement for travel-related quarantine. Further, this work was conducted with a 'real-time' with Public Health England to assist with the management of an active outbreak and inform immediate public health measures.

Reporting for specific materials, systems and methods

We require information from authors about some types of materials, experimental systems and methods used in many studies. Here, indicate whether each material, system or method listed is relevant to your study. If you are not sure if a list item applies to your research, read the appropriate section before selecting a response.

Materials & experimental systems

n/a	Involved in the study	
x		Antibodies
x		Eukaryotic cell lines
x		Palaeontology and archaeology
x		Animals and other organisms
x		Human research participants
x		Clinical data
x		Dual use research of concern

Methods

n/a	Involved in the study	
x		ChIP-seq
x		Flow cytometry
x		MRI-based neuroimaging
